# Out-of-sync evolutionary patterns and mutual interplay of major and minor capsid proteins in norovirus GII.2

**DOI:** 10.1099/jgv.0.002024

**Published:** 2024-09-27

**Authors:** Ruiquan Xu, Liang Xue, Jingmin Wang, Yiqing Chen, Yingwen Cao, Junshan Gao, Hui Gao, Qingyao Du, Xiaoxia Kou, Lin Yu

**Affiliations:** 1 Guangzhou Key Laboratory for Clinical Rapid Diagnosis and Early Warning of Infectious Diseases, KingMed School of Laboratory Medicine, Guangzhou Medical University, Guangzhou, PR China; 2 Department of Laboratory, The Key Laboratory of Advanced Interdisciplinary Studies Center, The First Affiliated Hospital of Guangzhou Medical University, Guangzhou, Guangdong, PR China; 3 Guangdong Provincial Key Laboratory of Microbial Safety and Health, State Key Laboratory of Applied Microbiology Southern China, Institute of Microbiology, Guangdong Academy of Sciences, Key Laboratory of Agricultural Microbiomics and Precision Application, Ministry of Agriculture and Rural Affairs, Guangzhou, Guangdong, 510070, PR China

**Keywords:** GII.2, interaction, minor capsid protein VP2, norovirus

## Abstract

Human noroviruses are the most common cause of viral gastroenteritis, resulting annually in 219 000 deaths and a societal cost of $60 billion, and no antivirals or vaccines are available. The minor capsid protein may play a significant role in the evolution of norovirus. GII.4 is the predominant genotype of norovirus, and its VP2 undergoes epochal co-evolution with the major capsid protein VP1. Since the sudden emergence of norovirus GII.2[P16] in 2016, it has consistently remained a significant epidemic strain in recent years. In the construction of phylogenetic trees, the phylogenetic trees of VP2 closely parallel those of VP1 due to the shared tree topology of both proteins. To investigate the interaction patterns between the major and minor capsid proteins of norovirus GII.2, we chose five representative strains of GII.2 norovirus and investigated their evolutionary patterns using a yeast two-hybrid experiment. Our study shows VP1–VP2 interaction in GII.2, with critical interaction sites at 167–178 and 184–186 in the highly variable region. In the intra-within GII.2, we observed no temporal co-evolution between VP1 and VP2 of GII.2. Notable distinctions were observed in the interaction intensity of VP2 among inter-genotype (*P*<0.05), highlighting the divergent evolutionary patterns of VP2 within different norovirus genotypes. In summary, the interactions between VP2 and VP1 of GII.2 norovirus exhibit out-of-sync evolutionary patterns. This study offered valuable insights for further understanding and completing the evolutionary mechanism of norovirus.

## Introduction

Human noroviruses (HuNoVs) are recognized as a major cause of acute gastroenteritis in all age groups [1]. Children, the elderly and individuals with compromised immune systems are particularly susceptible to experiencing severe HuNoV infections. Annually, approximately 700 million individuals contract an HuNoV infection, resulting in roughly 219 000 fatalities [2]. As HuNoV is highly contagious, outbreaks are difficult to control, and no approved antivirals or vaccines are currently available [3]. Furthermore, a significant annual economic burden is linked to HuNoV infections, amounting to $60 billion [2].

Norovirus (NoV) is a non-enveloped, positive-sense RNA virus in the calicivirus. NoVs have genomes ranging from 7.4 to 7.7 kb, containing three ORFs [4]. ORF2 encodes the major capsid protein (VP1), and ORF3 encodes the minor capsid protein (VP2). NoV exhibits significant genetic diversity and heterogeneity, falling into ten genogroups, namely, GI through GX [5]. Of these genogroups, GI, GII and GIV are known to infect humans, with each of them containing numerous genotypes [6]. NoV’s genetic diversity complicates predicting its epidemic trends. The development of an NoV vaccine has faced challenges due to the virus’s high variability and the persistent absence of reliable cellular and animal models [7]. Therefore, the study of virus evolution mechanisms and epidemic prediction becomes especially crucial.

Since the first worldwide pandemic of NoV in 1995–1996, NoV GII.4 has been epidemic and dominant worldwide, causing 50%–70% of NoV outbreaks each year [8]. During the winter of 2014–2015, the previously rare NoV GII.17[P17] strain emerged and replaced NoV GII.4 Sydney as the dominant genotype, threatening many areas in Asia [9]. NoV GII.2 was a rarely detected genotype before 2016, with only limited cases reported. The recombinant strain GII.2[P16] of NoV suddenly reappeared in the winter of 2016–2017 in China, Japan, Germany, France and the USA and has continued to circulate globally as a significant epidemic strain in recent times [10]. The TransPhylo analysis revealed the initiation of widespread transmission of NoV GII.2[P16] in 2010. Before this time frame, NoV GII.2[P16] had been prevalent in Japan, although its prevalence was constrained to a limited scale [11].

Variations in the sequence and structure of VP1 are associated with receptor binding, evading neutralizing antibodies and contributing to strain diversity [12]. Unlike VP1, VP2 also has a unique impact on the life cycle and evolution of NoV. The VP2 of NoV serves functions such as virus particle stabilization and negatively regulating the activity of RNA-dependent RNA polymerase (RRP) [13,14]. The co-expression of VP1 and VP2, in comparison to their individual expression, enhances the overall protein expression and facilitates the nuclear entry of VP1 [15]. In our prior research, we observed the interaction between VP2 and VP1 of NoVs GII.4 and GII.17 [16,17]. Notably, VP1 and VP2 of GII.4 exhibited a time-ordered co-evolution pattern, whereas VP2 and VP1 of GII.17 did not display such epochal evolutionary dynamics. Furthermore, the interaction domain on VP2 is situated within a highly variable region, and the evolutionary rate of the region encoding VP2 surpasses that of the region encoding VP1. Due to the various genetic diversities of HuNoV, the underlying cause for the sudden surge in the prevalence of uncommon strains has not been comprehensively examined. The prevalence of different genogroups or genotypes is significantly different, but the underlying mechanism is still unclear. Previous studies strongly support VP1–VP2 interaction, indicating that VP2’s evolutionary mechanism may be influenced by this interaction. This article explores VP1–VP2 interaction in GII.2 variants over the past 30 years. We identified crucial interaction sites in the highly variable VP2 region, shedding light on the evolutionary relationship and pattern of VP2 and VP1 in GII.2.

## Methods

### Data collection and analysis

We collected full-length nucleotide sequences of the GII.2 ORF2 and ORF3 genes from GenBank. These sequences include information about accession numbers, geographic regions and collection dates.

### Phylogenetic and evolutionary rate analyses

CD-HIT software was used to minimize the number of high-similarity sequences with a threshold of 0.99 [18]. The available data were aligned using MAFFT software v7.313 [19]. ModelFinder was used to select the best-fit model using Bayesian Information Criterion for the selected ORF2 and ORF3 genes [20]. The trees were constructed using MrBayes software 3.2.6 according to the best substitution model. The Markov Chain Monte Carlo (MCMC) runs were conducted with chain lengths of 9 000 000 steps, with sampling every 1000 steps for the selected sequences until convergence. The trees were visualized in the International Tree of Life (http://itol.embl.de/).

Evolutionary rates were estimated using a Bayesian Markov chain Monte Carlo (BMCMC) method within BEAST version 1.10.4 [21]. The mean evolutionary rate and the 95% upper and lower highest posterior density intervals were deduced from the posterior tree distribution generated through the BMCMC runs using Tracer version 1.7.2 [22].

### Yeast two-hybrid gold assays

#### Plasmid construction

Bait (pGBKT7) and prey (pGADT7) plasmids contain different full lengths of VP2 and VP1 of the selected GII.2 strains based on previous retrieves. The sequences, including SMV1982 (accession: AY134748), Tokyo2004 (accession: DQ456824), Ho2011 (accession: LC122833), GZ2015 (accession: MK729086) and BJSMQ2016 (accession: NC_039476), were amplified using PrimeSTAR polymerase (Takara, Beijing, China). The recombinant plasmid primers are given in Table S1. Then, PCR fragments were inserted into the pGBKT7 and pGADT7 vectors using the Uniclone One Step Seamless Cloning Kit (Genesand Biotech, Beijing, China) to form plasmids.

#### Two-hybrid screening

The yeast two-hybrid assay was conducted following the guidelines outlined in the Yeast Protocols Handbook of Clontech. Yeast competent cells were prepared using the LiAc/PEG3350-based method according to the Yeastmaker Yeast Transformation System 2 User Manual. The recombinant plasmids pGBKT7-VP2 were introduced into the yeast competent cells using carrier DNA and 1.1× PEG/LiAC at 30°C for 30 min; then, DMSO was added at 42°C for 15 min. The yeast containing recombinant plasmids pGBKT7-VP2 were spread onto agar plates with synthetic drop-out medium lacking tryptophan (SD/Trp) for incubation at 30°C until colonies appeared.

Subsequently, colonies were screened for autoactivation (growth on SD/-Trp/-Ade/-His) and toxicity (36-h growth curve assay). Following the confirmation, well-checked transformants were used to generate competent cells. Then, recombinant plasmids pGADT7-VP1 were transformed into competent cells and incubated at 30℃. Blue colonies grown on plates with medium SD/-Trp/-Leu/Ade/-His/X-gal were positive interactions. Positive interaction colonies were cultured and assayed for α-galactosidase activity according to the manufacturer’s instruction. The *t*-test was used to analyse the data, and *P*<0.05 is considered statistically significant.

## Result

### Evolutionary and epidemic analyses of GII.2 variants

**Fig. 1.**
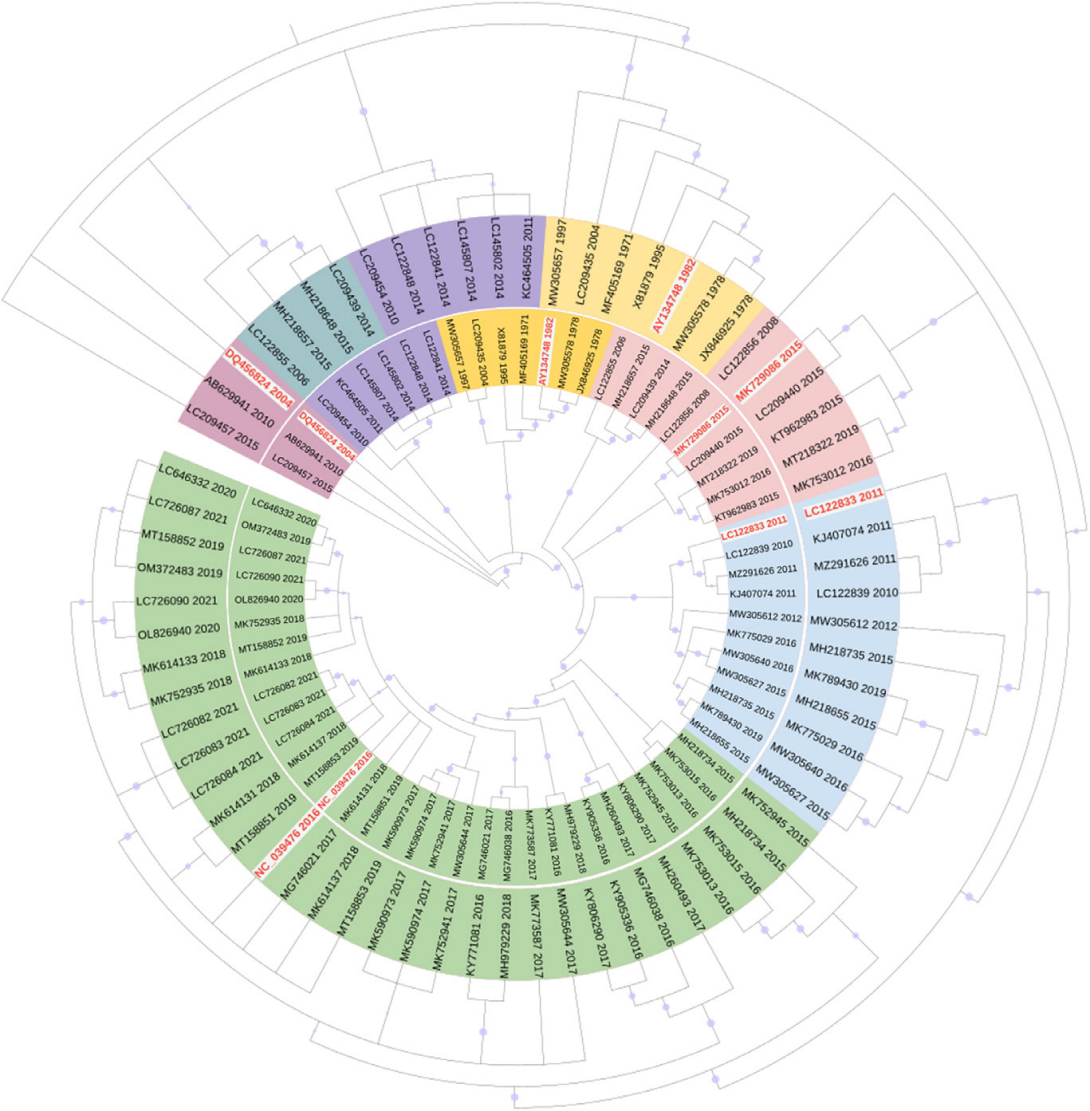
Phylogenetic trees of the NoV GII.2 VP1 and VP2 genes. The outer tree was constructed using GII.2 VP1 nucleotide sequences, while the inner tree represents the GII.2 VP2 phylogenetic tree. These trees were constructed using MrBayes software 3.2.6 and visualized in the International Tree of Life (iTOL, http://itol.embl.de/). Various colours were used to differentiate between clades. Representative strains selected for this experiment are indicated in red text.

A total of 319 sequences, including both VP1 and VP2 genes of NoV GII.2, were collected up to March 2023. CD-HIT software was used to minimize the number of high-similarity sequences with a threshold of 0.99. After screening, 69 sequences remained for the evolutionary analysis (accession numbers in Table S1, available in the online version of this article). The phylogenetic tree was constructed using the Bayesian information criterion (GTR+G). The phylogenetic trees of the VP1 and VP2 genes in GII.2 exhibit similar topologies, with each branch roughly corresponding to the same year (Fig. 1). The evolutionary rate of the VP2 gene is 2.786×10^−3^ substitutions/site/year, which is close to those in the VP1 region (3.393×10^−3^ substitutions/site/year). We prioritize clinical samples that have been tested and preserved in our laboratory. Subsequently, representative strains are selected based on diverse evolutionary branches, emphasizing those with extended circulation time and numerous clusters. Five representative strains, namely, AY134748, DQ456824, LC122833, MK729086 and NC_039476, have been selected and are listed in Table 1. In addition, scanning nucleotide similarity analysis identified a hypervariable region in VP2 of these GII.2 variants, and the nucleotide similarity decreased to nearly 75% (Fig. S1).

**Table 1.** Genetic information of five representative viruses

GenBank accession	Strain	Genotype	Named	Comment
AY134748	Snow mountain virus	GII.2[P30]	SMV1982	Ancestral strain
DQ456824	Hu/MK04/2004/JP	GII.2[P2]	Tokyo2004	Appeared earlier in the cluster
LC122833	Hu/GII/JP/2011/GII.P16-GII.2/HO-17	GII.2[P16]	Ho2011	Appeared earlier in the cluster
MK729086	Hu/Guangzhou/GZ2015-L335/CHN/2015	GII.2[P2]	GZ2015	Lab preserved
NC_039476	Env/CHN/2016/GII.2[P16] BJSMQ	GII.2[P16]	BJSMQ2016	Reference strain

### Autoactivation and toxicity testing

**Fig. 2.**
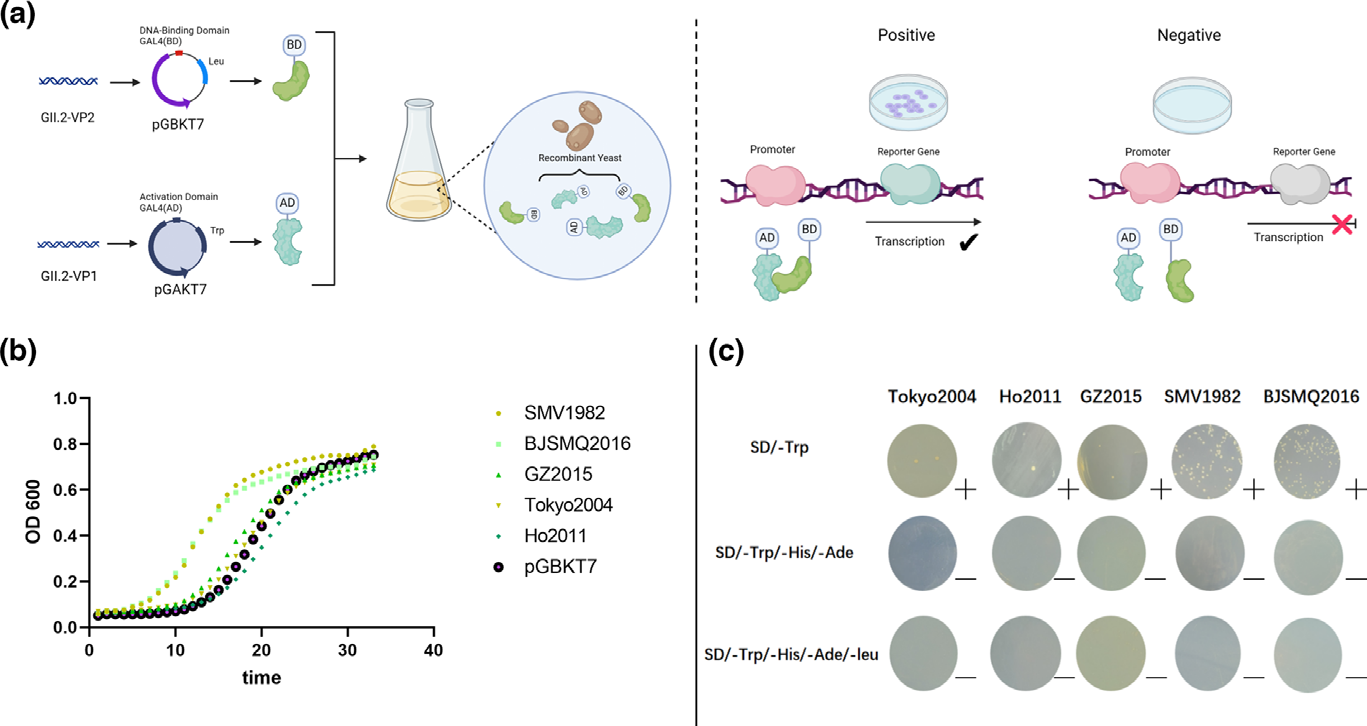
(**a**) Illustration of the yeast two-hybrid assay. Two proteins to be tested for interaction were expressed in yeast as hybrid fusion proteins. VP2 was fused into pGBKT7 (DNA-binding domain), and VP1 was fused into pGADT7 (activation domain). The reporter genes will be activated when these two proteins interact, forming colonies on agar plates with medium SD/-Trp/-Leu/-His/-Ade/ X. (**b**) The toxicity tests of the VP2 proteins. Plasmid pGBKT7 was set as a control group at the same time. All five VP2 proteins did not hinder Y2H Gold yeast growth when compared to the control group. (**c**) VP2 proteins could not grow in the minus medium and could not activate reporter gene expression in Y2H Gold yeast. Compared with the control group, these proteins did not inhibit the growth of yeast. The symbols on the right: ‘+’ means colony growth and ‘−’ means no growth.

The Y2H Gold yeast transformed with pGBKT7-GII.2 VP2 and pGBKT7 empty plasmid exhibited robust growth on SD/-Trp plates but failed to grow on SD/-Trp/-Ade/-His culture media. The results of autoactivation testing indicated that VP2 could not activate reporter gene expression in Y2H Gold yeast (Fig. 2c). There was no significant difference between the experimental and control groups in terms of the number and morphology of yeast colonies. Toxicity testing (36-h growth curve assay) confirmed that these proteins did not hinder the growth of yeast (Fig. 2b). These findings support the suitability of the yeast two-hybrid system for investigating the interaction between VP1 and VP2.

### Interaction between VP1 and VP2 of GII.2 variants

**Fig. 3.**
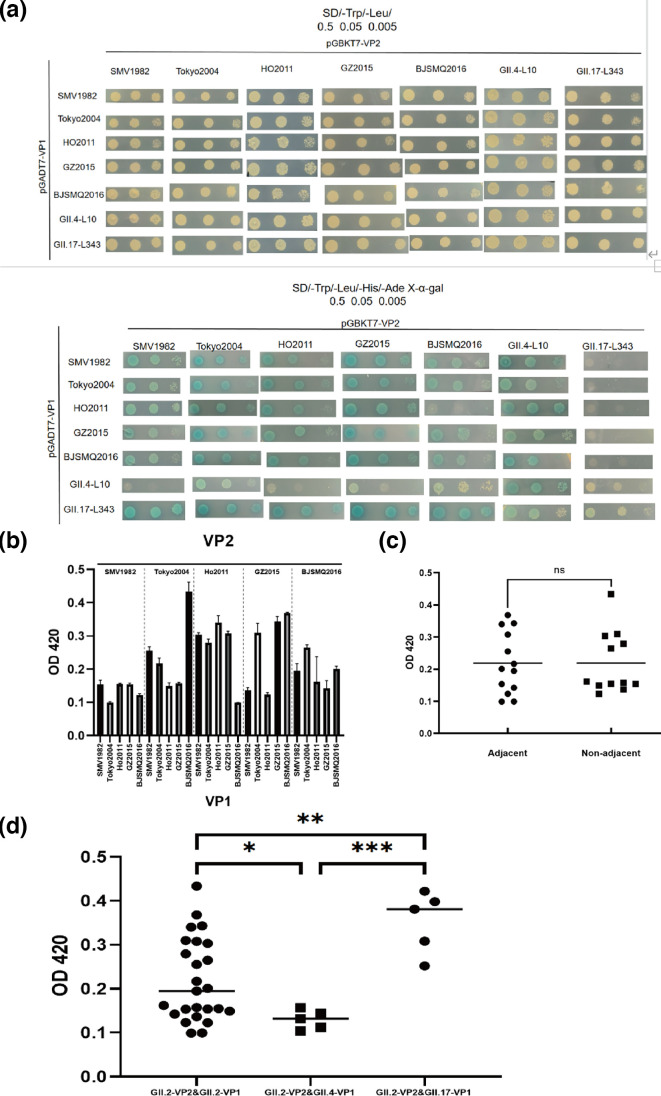
Quantitative assessment of the interaction strength between VP1 and VP2 of NoV. (**a**) The growth of recombinant yeast containing two plasmids was assessed on SD/-Trp/-Leu and SD/-Trp/-Leu/-Ade-/His plates. (**b**) Each VP1 interacts with VP2 among pandemic GII.2 variants in the quantitative α-galactosidase activity assay. Error bars denote sds. (**c**) Quantitative α-galactosidase activity assay of the adjacent and non-adjacent VP1/VP2 interaction. (**d**) Inter-genotype experiments involved the quantitative detection of the interaction of GII.2 VP2 with VP1 from GII.2, GII.4 and GII.17, respectively.

We created recombinant plasmids pGADT7-VP1 and introduced them into Y2H Gold yeast competent cells containing pGBKT7-VP2 recombinant plasmids. Positive interactions were verified by the presence of single colonies on SD/-Trp/-Leu/-Ade/-His plates. Fig. 3a, b illustrates the interactions among five GII.2 variants. Furthermore, we conducted inter-genotype experiments between the major and minor capsid proteins of GII.4 and GII.17. Although interactions were exhibited among all variants, variations were observed during culturing, such as differences in colony size and number, indicating the potential differences in the strength of interactions. Furthermore, a quantitative α-galactosidase assay was used to assess their interaction strength. Based on the large-scale transmission of GII.2 starting in 2010, we speculate that the transmission capacity of GII.2 was strengthened in 2010, marking a crucial time point for distinguishing the evolution of GII.2. Therefore, Ho2011 was taken as the dividing point, and these variants were divided into two periods according to the epidemic time. SMV1982 and Tokyo2004 belong to one period, and Ho2011, GZ2015 and BJSMQ2016 belong to another period. VP1 and VP2 interaction intensities within the same periods are termed adjacent, while others are considered non-adjacent. For example, the interaction between SMV1982’s VP1 and Tokyo2004’s VP2 would be considered an adjacent group, while the interaction between SMV1982’s VP1 and Ho2011’s VP2 would be classified as a non-adjacent group. In Fig. 3c, the interactions between GII.2 VP1 and VP2 are divided into two groups: adjacent and non-adjacent groups based on time. We observed no significant difference in the interaction strength between the two groups, suggesting that there is no trend of co-evolution over time. In Fig. 3d, the interaction strengths of GII.2 NoV VP2 with VP1 of GII.2, GII.4 and GII.17 are detected. We found a significant variation among the three sets of data, indicating that the capsid proteins of these three genotypes have distinct evolutionary patterns.

### Identification of the key domain on VP2 for its interaction with VP1

**Fig. 4.**
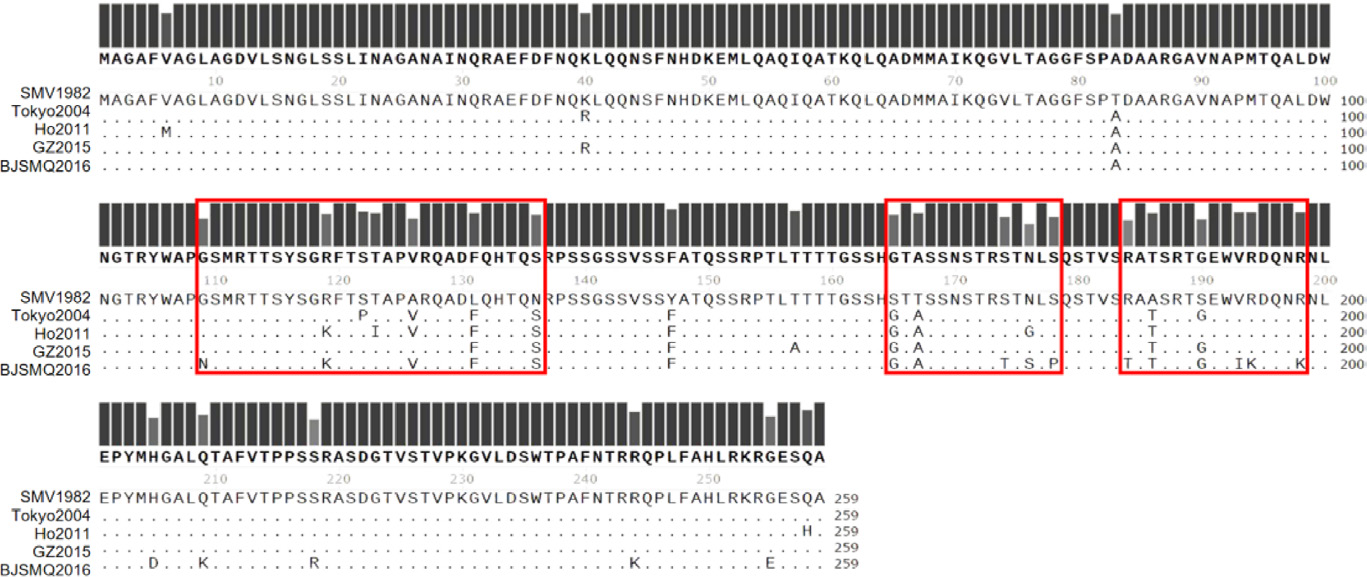
Amino acid alignment of five variants. Residues with the highly variable region across the five variants are highlighted within a red box. The protein sequence of SMV1982 in the first row serves as the reference sequence. The black dots in rows 4–5 indicate identical aas, while other letters represent differing aas.

**Fig. 5.**
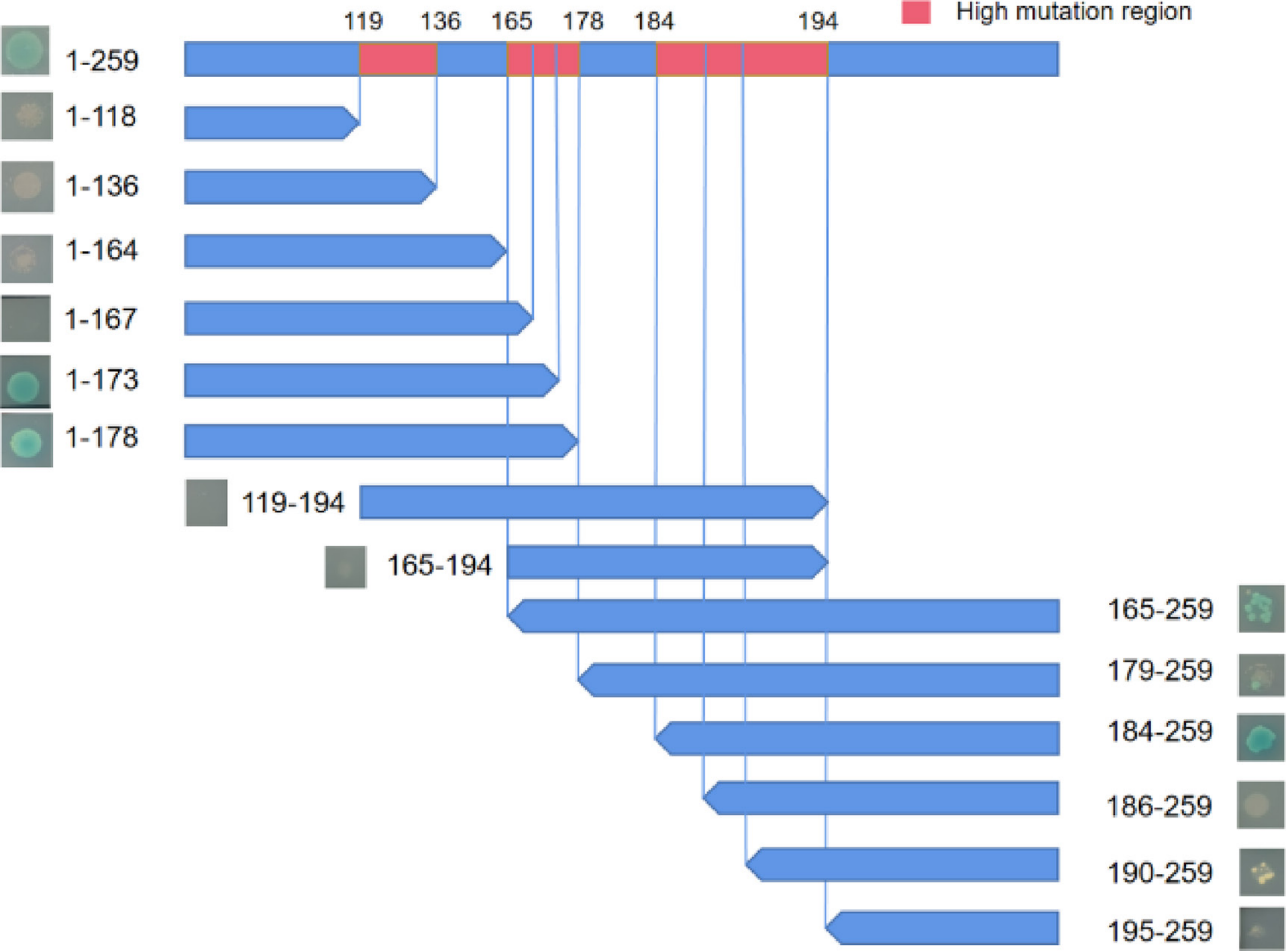
Diagram of residue mutants and interaction presentation. The presence of a single colony implies the presence of interaction. The red box highlights the high mutation region of VP2. Blue single colonies indicate the presence of positive interactions.

Previous research has indicated that the hypervariable region on VP2 plays an important role in interactions. We compared the VP2 aa sequences of five variants and identified three regions with high aa variability, namely, 119–136, 165–178 and 184–194 (Fig. 4). To investigate whether the three hypervariable regions of VP2 are crucial for these interactions, we designed a series of truncated mutants for VP2. Specific primers were created for truncated VP2, resulting in the construction of recombinant plasmids: 1–118, 1–136, 1–164, 1–167, 1–173, 1–178, 165–259, 179–259, 184–259, 186–259, 190–259 and 194–259 (Fig. 5).

The full-length VP2 protein and mutants containing aa residues 1–173 and 1–178 have been observed to interact with VP1, whereas the mutant containing aa residues 1–167 does not exhibit this interaction. This underscores the involvement of residues 167–178 in the interaction with VP1. To further pinpoint the critical regions, a series of mutants with N-terminal residue deletions were generated, and their interactions with VP1 were assessed. Our findings indicate that mutants lacking the first 165 and 184 residues retain their ability to interact with VP1. However, the mutant lacking the first 179 residues shows a noticeable weakening of interaction strength, while the mutant lacking the first 186 residues completely loses its ability to interact with VP1. As a result, residues 184–186 are identified as crucial for the VP1 interaction, and residues 179–184 are capable of modulating the strength of this interaction.

Furthermore, mutants with deletions of residues before and after two specific segments, 119–194 and 165–194, were constructed and evaluated for their interactions with VP1. The results suggest that fragments lacking residues before and after these two segments do not engage in interactions with VP1. This leads to the inference that the presence of N-terminal and C-terminal residues of the highly variable region of VP2 is essential for the VP2–VP1 interaction.

## Discussion

NoV GII.2 was a rarely detected genotype before 2016, with only limited cases reported. This genotype suddenly reappeared and became the main genotype in China and other countries, holding prominence as the predominant epidemic variant during the years 2016–2017 [11]. Our interest lies in understanding the abrupt rise of GII.2, non-GII.4 genotypes. Understanding the interaction between major and minor capsid proteins of NoV is essential for unravelling the epidemic mechanisms of diverse HuNoV strains. Our findings reveal an interaction between the VP1 and VP2 of GII.2, with the crucial VP2 interaction sites 167–178 and 184–186 situated within the highly variable region. Notable distinctions were observed in the interaction intensity of VP2 among inter-genotypes (*P*<0.05), highlighting the divergent evolutionary patterns of VP2 within different NoV genotypes.

In our earlier research, we examined the interplay between major and minor capsid proteins in both GII.4 and GII.17, revealing distinct patterns in intra-genotype interaction and crucial sites. On the other hand, in the biological functioning of proteins, interactions with other proteins are frequently integral [23]. Investigating the interaction of capsid proteins among various genotypes offers the essential groundwork for understanding virus mechanisms and conducting evolutionary analyses. Based on these foundations, we want to further explore the intra-genotype interactions within GII.2 and inter-genotype interactions between GII.2 and other prevalent strains. Interestingly, there are robust interactions between VP2 of GII.4 and all VP1 of GII.2. However, the interaction intensity of GII.17 VP2 with other genotypes’ VP1 is notably weak. In the inter-genotype interaction, proteins within the same gene group exhibit a stronger interaction compared to those in different gene groups. Notably, the VP1 of GII.17 exhibits limited protein interaction with other strains. Distinct genotypes possess varying conserved sites, underscoring the need for a more in-depth exploration of the crucial aa sites involved in protein interaction. This analysis is essential for understanding the mechanism of protein interaction. The prolonged prevalence and ongoing evolution of dominant strains lead to certain genotypes following distinct evolutionary paths. This divergence could explain the differences in the prevalence compared to other non-epidemic genotypes. NoV GII.4 manifests sustained prevalence, and during co-evolutionary processes, the VP2 protein has been shown to significantly enhance the structural stability and infectivity of the virus. However, in other non-GII.4 genotypes of NoV, VP2 may compromise the virus’s overall stability.

In GII.4, the crucial interaction domain for VP2 involves residues 131–160 and 171–180, whereas in GII.17, it involves residues 174–179 [17]. In GII.2, aa residues 167–178 and 184–186 on VP2 are identified as pivotal regions for the interaction. Therefore, examining the evolutionary patterns and interactions of VP1 and VP2 in various epidemic strains helps enhance our understanding of viral evolutionary mechanisms. Among these, residues 167–178 exhibit a higher degree of variability in comparison to residues 184–186. Importantly, non-hypervariable regions of VP2, residues 179–183, exert an inhibitory effect on the interaction with the major capsid protein. Residues 119–194 and 165–194 cannot interact with VP1, highlighting the necessity of N-terminal or C-terminal residues of the highly variable region of VP2 for the VP2–VP1 interaction. It may influence VP2’s ability to fold into its normal spatial structure. VP2’s key interaction domain can adopt the correct structure for binding to VP1 only when the terminal residues are present.

To investigate the impact of the interaction domain on the evolution of VP2, we compared their phylogenetic tree. Interestingly, the branches of the phylogenetic tree of VP1 and VP2 are close [24], but the nucleic acid evolution of the interaction domain of VP2 is significantly different from that of VP1. Consequently, VP2 and VP1 of GII.2 NoV have not exhibited co-evolution in their interactions over the past 30 years. The similarity in evolutionary branching patterns between VP1 and VP2 may be attributed to their relatively conservative variability. It shows that the degree of variation of VP1 and VP2 of GII.2 is very limited in these 30 years. The observed difference in co-evolution between VP1 and VP2 in GII.2 compared to GII.4 may be attributed to the fast mutation rate of GII.4. The high variability spanning 50 years in GII.4 allows for the co-evolutionary accumulation of VP2 and VP1. In contrast, GII.2 does not demonstrate a distinct co-evolutionary pattern. As GII.2 becomes the predominant epidemic variant, enhancing detection and sequence analysis of its VP2 is crucial for tracking NoV evolution.

In recent years, numerous scientists have conducted extensive research to understand the factors contributing to the sudden prevalence of GII.2. Minor proteins have received a lot of attention. According to the biological structure observation of feline calicivirus, the minor protein can form a porous structure on the surface of the receptor and assist the process of viral nucleic acid entering the cell [25]. Our research provides evidence indicating that the molecular mechanism behind its outbreak may not be linked to the more conserved capsid protein. Amino acid mutations in non-structural proteins of the virus, particularly RDRP, could potentially be the molecular mechanism driving the outbreak of the NoV [26]. Furthermore, it is suggested that the RDRP of GII.2[P16] exhibits improved heat resistance, a feature that may contribute to the prominence of this strain amid ongoing global warming [27]. In short, the reasons behind new non-GII.4 NoV outbreaks are likely diverse. Analysing the molecular mechanism behind the GII.2 outbreak requires a fresh perspective or a more comprehensive examination, possibly involving the interaction of ORF 1–3.

## supplementary material

10.1099/jgv.0.002024 Uncited Fig. S1.

10.1099/jgv.0.002024 Uncited Table S1.

## Abbreviations

BMCMC: Bayesian Markov chain Monte Carlo
HuNoV: human norovirus
NoV: norovirus
RDRP: RNA-dependent RNA polymerase
SD/Trp: synthetic drop-out medium lacking tryptophan

## References

[1] Quee FA, de Hoog MLA, Schuurman R, Bruijning-Verhagen P (2020). Community burden and transmission of acute gastroenteritis caused by norovirus and rotavirus in the Netherlands (RotaFam): a prospective household-based cohort study. Lancet Infect Dis.

[2] Bartsch SM, Lopman BA, Ozawa S, Hall AJ, Lee BY (2016). Global economic burden of norovirus gastroenteritis. PLoS One.

[3] Tan M, Jiang X (2019). Norovirus capsid protein-derived nanoparticles and polymers as versatile platforms for antigen presentation and vaccine development. Pharmaceutics.

[4] Hardy ME (2005). Norovirus protein structure and function. FEMS Microbiol Lett.

[5] Chhabra P, de Graaf M, Parra GI, Chan MC-W, Green K (2019). Updated classification of norovirus genogroups and genotypes. J Gen Virol.

[6] Vinjé J (2015). Advances in laboratory methods for detection and typing of norovirus. J Clin Microbiol.

[7] Morales-Hernández S, Ugidos-Damboriena N, López-Sagaseta J (2022). Self-assembling protein nanoparticles in the design of vaccines: 2022 update. Vaccines.

[8] Tohma K, Lepore CJ, Gao Y, Ford-Siltz LA, Parra GI (2019). Population genomics of GII.4 noroviruses reveal complex diversification and new antigenic sites involved in the emergence of pandemic strains. mBio.

[9] Chan MCW, Lee N, Hung T-N, Kwok K, Cheung K (2015). Rapid emergence and predominance of a broadly recognizing and fast-evolving norovirus GII.17 variant in late 2014. Nat Commun.

[10] Shen W, Sheng Y, Weng J, Li G, Wang D (2020). Molecular epidemiology of norovirus associated with acute gastroenteritis in Taizhou, China: a retrospective study. J Infect Public Health.

[11] Wang L, Ji L, Li H, Xu D, Chen L (2022). Early evolution and transmission of GII.P16-GII.2 norovirus in China. G3 Genes|Genom|Genet.

[12] Cotten M, Petrova V, Phan MVT, Rabaa MA, Watson SJ (2014). Deep sequencing of norovirus genomes defines evolutionary patterns in an urban tropical setting. J Virol.

[13] Lin Y, Fengling L, Lianzhu W, Yuxiu Z, Yanhua J (2014). Function of VP2 protein in the stability of the secondary structure of virus-like particles of genogroup II norovirus at different pH levels: function of VP2 protein in the stability of NoV VLPs. J Microbiol.

[14] Subba-Reddy CV, Goodfellow I, Kao CC (2011). VPg-primed RNA synthesis of norovirus RNA-dependent RNA polymerases by using a novel cell-based assay. J Virol.

[15] Liu Z, Zhang M, Shen Z, Chen H, Zhang W (2019). The coordinating role of the human norovirus minor capsid protein VP2 is essential to functional change and nuclear localization of the major capsid protein VP1. Arch Virol.

[16] Liao Y, Wang L, Hong X, Gao J, Zuo Y (2022). The VP2 protein exhibits cross-interaction to the VP1 protein in norovirus GII.17. Infect Genet Evol.

[17] Hong X, Xue L, Gao J, Jiang Y, Kou X (2022). Epochal coevolution of minor capsid protein in norovirus GII.4 variants with major capsid protein based on their interactions over the last five decades. Virus Res.

[18] Fu L, Niu B, Zhu Z, Wu S, Li W (2012). CD-HIT: accelerated for clustering the next-generation sequencing data. *Bioinformatics*.

[19] Katoh K, Standley DM (2013). MAFFT multiple sequence alignment software version 7: improvements in performance and usability. Mol Biol Evol.

[20] Kalyaanamoorthy S, Minh BQ, Wong TKF, von Haeseler A, Jermiin LS (2017). ModelFinder: fast model selection for accurate phylogenetic estimates. Nat Methods.

[21] Suchard MA, Lemey P, Baele G, Ayres DL, Drummond AJ (2018). Bayesian phylogenetic and phylodynamic data integration using BEAST 1.10. Virus Evol.

[22] Rambaut A, Drummond AJ, Xie D, Baele G, Suchard MA (2018). Posterior summarization in Bayesian phylogenetics using tracer 1.7. Syst Biol.

[23] Valtonen S, Vuorinen E, Kariniemi T, Eskonen V, Le Quesne J (2020). Nanomolar protein-protein interaction monitoring with a label-free protein-probe technique. Anal Chem.

[24] Tohma K, Lepore CJ, Ford-Siltz LA, Parra GI (2017). Phylogenetic analyses suggest that factors other than the capsid protein play a role in the epidemic potential of GII.2 norovirus. mSphere.

[25] Conley MJ, McElwee M, Azmi L, Gabrielsen M, Byron O (2019). Calicivirus VP2 forms a portal-like assembly following receptor engagement. Nature.

[26] Ao Y, Cong X, Jin M, Sun X, Wei X (2018). Genetic analysis of reemerging GII.P16-GII.2 noroviruses in 2016-2017 in China. J Infect Dis.

[27] Tan MTH, Xue L, Wang D, Eshaghi Gorji M, Li Y (2022). The globally re-emerging norovirus GII.2 manifests higher heat resistance than norovirus GII.4 and Tulane virus. J Appl Microbiol.

