# Erratum: Out-of-sync evolutionary patterns and mutual interplay of major and minor capsid proteins in norovirus GII.2

**DOI:** 10.1099/jgv.0.002074

**Published:** 2025-02-03

**Authors:** Ruiquan Xu, Liang Xue, Jingmin Wang, Yiqing Chen, Yingwen Cao, Junshan Gao, Hui Gao, Qingyao Du, Xiaoxia Kou, Lin Yu

**Affiliations:** 1 Guangzhou Key Laboratory for Clinical Rapid Diagnosis and Early Warning of Infectious Diseases, KingMed School of Laboratory Medicine, Guangzhou Medical University, Guangzhou, PR China; 2 Department of Laboratory. The Key Laboratory of Advanced Interdisciplinary Studies Centre, The First Affiliated Hospital of Guangzhou Medical University, Guangzhou, Guangdong, PR China; 3 Guangdong Provincial Key Laboratory of Microbial Safety and Health, State Key Laboratory of Applied Microbiology Southern China, Institute of Microbiology, Guangdong Academy of Sciences, Key Laboratory of Agricultural Microbiomics and Precision Application, Ministry of Agriculture and Rural Affairs, Guangzhou, Guangdong, 510070, PR China

In the published version of this article, there were errors in the affiliation list. Authors Liang Xue and Junshan Gao should be linked to affiliation 3, and authors Ruiqan Xu and Lin Yu should be linked to affiliations 1 and 2.

Previously, the author and affiliation list appeared as below:

Ruiquan Xu^1^, Liang Xue^2^, Jingmin Wang^1^, Yiqing Chen^1^, Yingwen Cao^1^, Junshan Gao^2^, Hui Gao^1^, Qingyao Du^1^, Xiaoxia Kou^1^ and Lin Yu^1,3^

^1^Guangzhou Key Laboratory for Clinical Rapid Diagnosis and Early Warning of Infectious Diseases, KingMed School of Laboratory Medicine, Guangzhou Medical University, Guangzhou, PR China

^2^Department of Laboratory. The Key Laboratory of Advanced Interdisciplinary Studies Centre. The First Affiliated Hospital of Guangzhou Medical University, Guangzhou, Guangdong, PR china

^3^Guangdong Provincial Key Laboratory of Microbial Safety and Health, State Key Laboratory of Applied Microbiology Southern China, Institute of Microbiology, Guangdong Academy of Sciences, Key Laboratory of Agricultural Microbiomics and Precision Application, Ministry of Agriculture and Rural Affairs, Guangzhou, Guangdong, 510070, PR China

The correct author and affiliation list should have appeared as:

Ruiquan Xu^1,2^, Liang Xue^3^, Jingmin Wang^1^, Yiqing Chen^1^, Yingwen Cao^1^, Junshan Gao^3^, Hui Gao^1^, Qingyao Du^1^, Xiaoxia Kou^1^, Lin Yu^1,2^

^1^Guangzhou Key Laboratory for Clinical Rapid Diagnosis and Early Warning of Infectious Diseases, KingMed School of Laboratory Medicine, Guangzhou Medical University, Guangzhou, PR China

^2^Department of Laboratory. The Key Laboratory of Advanced Interdisciplinary Studies Centre. The First Affiliated Hospital of Guangzhou Medical University, Guangzhou, Guangdong, PR china

^3^Guangdong Provincial Key Laboratory of Microbial Safety and Health, State Key Laboratory of Applied Microbiology Southern China, Institute of Microbiology, Guangdong Academy of Sciences, Key Laboratory of Agricultural Microbiomics and Precision Application, Ministry of Agriculture and Rural Affairs, Guangzhou, Guangdong, 510070, PR China

The Microbiology Society apologizes for any inconvenience caused.

