# Supplementary material for: Out-of-sync evolutionary patterns and mutual interplay of major and minor capsid proteins in norovirus GII.2
Source: J Gen Virol. 2024 Sep 27;105(9):002024. doi: 10.1099/jgv.0.002024 (PMC11430271; doi:10.1099/jgv.0.002024)
Supplement: Uncited Fig. S1. [file jgv-105-02024-s001.pdf]

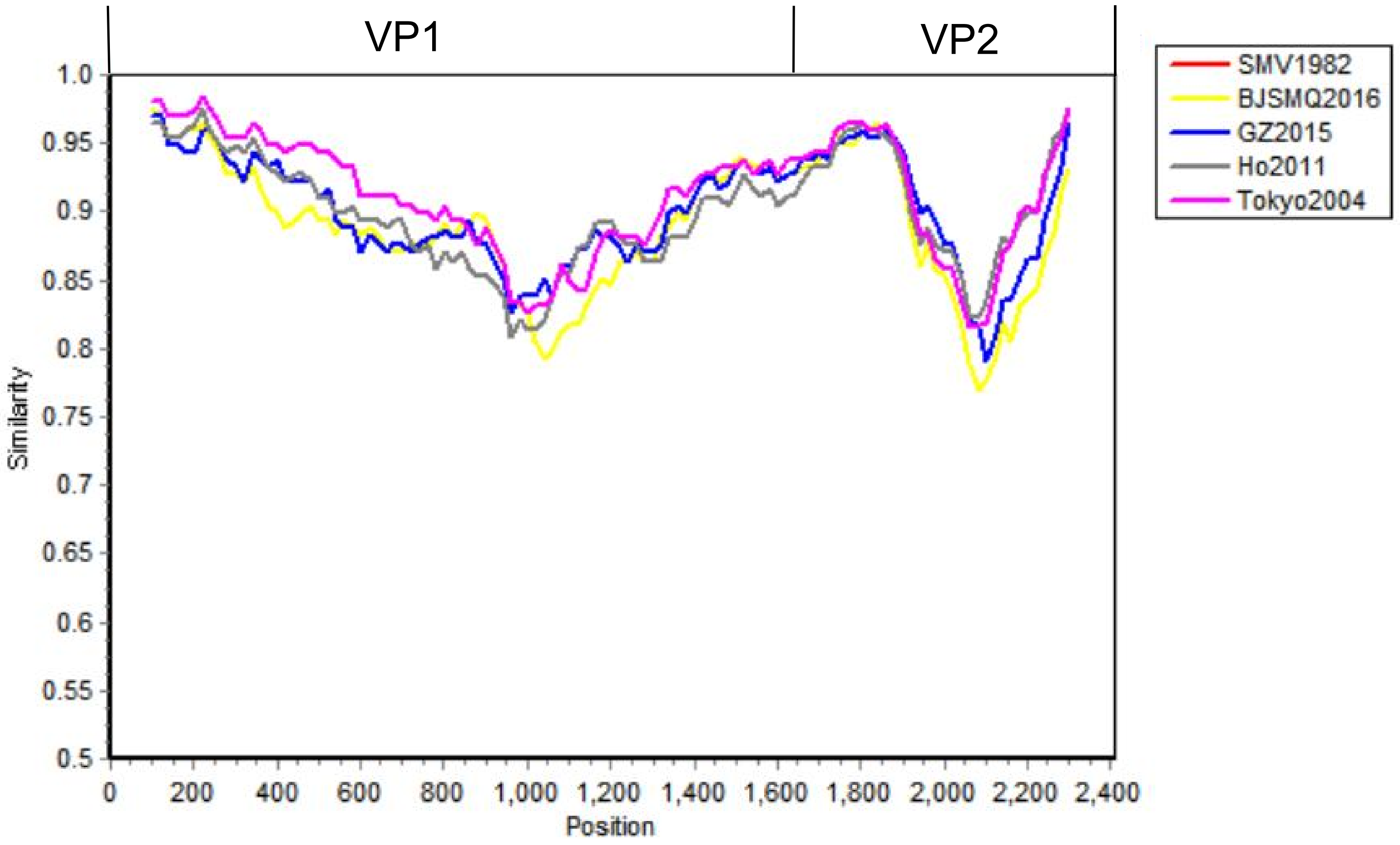

Supplemental Figure 1. Similarity plot of GII.2 variants. The graph represented as a percentage similarity of the five selected strains. The window size was 200 bp with a step size of 20 bp, and distance model was Kimura 2 parameter.
